# Impact of *Aggregatibacter actinomycetemcomitans* on inflammasome regulation and epigenetic changes in grade C periodontitis

**DOI:** 10.1007/s00784-026-07040-1

**Published:** 2026-07-23

**Authors:** Aurelio Amorim Reis, Hélvis E. S. Paz, Catharina Marques Sacramento, Karina Gonzales Silvério, Enilson Antonio Sallum, Mabelle de Freitas Monteiro, Renato C. V. Casarin, Camila Schmidt Stolf

**Affiliations:** 1 Department of Prosthodontics and Periodontics, Piracicaba Dental School, University of Campinas, Piracicaba, São Paulo, Brazil; 2 Department of Dental Clinics, Oral Pathology and Oral Surgery, Periodontology Division, School of Dentistry, Universidade Federal de Minas Gerais, Belo Horizonte, Brazil; 3 Department of Periodontics and Endodontics, School of Dental Medicine, University at Buffalo, Buffalo, NY USA

**Keywords:** Periodontitis, Inflammation, Epigenomics, Fibroblasts

## Abstract

**Objectives:**

The NLRP3 inflammasome amplifies inflammation and may contribute to the destruction of tooth-supporting tissues in periodontitis. This study evaluated the expression of inflammasome markers (NLRP3, AIM2, IL-1β, TNF-α, CASP1, IL-10, TLR2, and TLR4) and the epigenetic patterns of NLRP3 and IL-1β in primary gingival fibroblasts from healthy individuals and patients with Grade C periodontitis (PerioC).

**Materials and methods:**

Cells were exposed to *Aggregatibacter actinomycetemcomitans* Protein Extract (*Aa*PE) (5 µg/ml) for 1.5 h. After the experimental period, total RNA and DNA were collected and extracted. Total RNA was isolated and gene expressions of TLR2, TLR4, NLRP3, CASP1, AIM2, IL-10, IL-1β and TNF-α were analyzed via qPCR. DNA methylation and hydroxymethylation patterns of the IL-1β and NLRP3 genes were evaluated. Statistical analyses were performed considering a significance level of 5%.

**Results:**

Fibroblasts from PerioC exhibited higher AIM2 levels before and after stimulation compared to healthy individuals (*p* < 0.05). Conversely, IL-10 expression was lower in PerioC before *Aa*PE incubation (*p* < 0.05). *NLRP3* expression increased post-stimulation in healthy cells but remained unchanged in PerioC. IL-1β expression was consistently lower in PerioC in both conditions. The NLRP3 gene showed reduced methylation after *Aa*PE exposure only in healthy fibroblasts, resulting in lower methylation levels compared to PerioC (*p* < 0.05).

**Conclusions:**

PerioC fibroblasts exhibited dysregulated inflammasome-related gene expression and a distinct epigenetic response to AaPE, suggesting altered local immune regulation at the transcriptional level.

**Clinical relevance:**

Patients with severe and rapidly progressing periodontitis exhibit an altered inflammasome-related gene expression and epigenetic response to bacterial stimulation. The reduced transcriptional responsiveness of inflammatory mediators and distinct DNA methylation profile observed in PerioC cells may contribute to ineffective host defense and persistent periodontal tissue destruction.

## Introduction

Severe (Stages III-IV) Grade C Periodontitis (PerioC) affecting young individuals is an inflammatory disease characterized by accelerated and severe bone loss in systemically healthy subjects [1, 2]. Studies have elucidated strong associations between microbiological and immunological characteristics with this disease, including alterations in the production of pro-inflammatory cytokines and a specific microbial profile, notably elevated levels of *Aggregatibacter actinomycetemcomitans* (*Aa*) [3–7]. Cytokine production is initiated on cell surfaces, where membrane receptors (Toll-like receptors) recognize microbial patterns, such as lipopolysaccharide (LPS) [8, 9]. Toll-like receptors, mainly Toll-like 2 and Toll-like 4 [10–12], are well-documented as receptors for recognizing specific pathogens such as viruses, bacteria, fungal species, and outer membrane components of Gram-negative bacteria. This recognition triggers intracellular cascades resulting in cytokine and active molecule production [13, 14].

Ongoing investigations seek to establish connections between periodontal destruction and the inflammasome [15], a complex of innate immune system multiprotein sensors responsible for activating caspases and inducing a range of inflammatory disturbances, often implicated in the onset or progression of diseases with significant public health impact [16, 17]. Recent studies understand antigen recognition by Toll-like receptors [10], encompassing recognition of pathogen-associated molecular patterns (PAMPs) [18–20], and danger-associated molecular patterns (DAMPs), the latter induced by endogenous stress via pattern recognition receptors (PRRs) [21]. Activation of these receptors triggers signaling cascades, culminating in type 1 interferon (α and β) and cytokine production [22–24]. The PRR family plays a pivotal role in the inflammasome complex, featuring nucleotide-binding domains, leucine-rich repeat proteins (NOD-like, NLRs) [25], and absent in melanoma 2 (AIM2) receptors. These domains activate caspase-1 through oligomerization, promoting the maturation of the pro-inflammatory cytokines IL-1β and IL-18 into their bioactive forms and contributing to pyroptosis, an inflammatory form of programmed cell death [26, 27].

Recent investigations have unveiled a direct link between periodontal disease and the multiprotein inflammasome complex [15–17, 20, 28–30]. The production of a specific cytokine pattern is directly tied to the inflammasome [13, 22, 23], as monocytes/macrophages exhibit heightened sensitivity to this leukotoxin, resulting in substantial IL-1β and cytokine levels before target cells undergo pyroptosis [22, 28]. This connection establishes inflammasome activation and IL-1β production, a molecule associated with various inflammatory diseases. Conversely, the intracellular complex can be activated by IL-1β, IL-10, and IL-18 [22]. The expression of NLRP3 is markedly implicated in the inflammasome in patients with chronic and aggressive periodontitis compared to healthy subjects [15, 31]. Given the increased importance of inflammasome in periodontal disease etiopathogenesis and the relatively few studies in cells from affected patients, this study aims to investigate the gene expression of inflammasome markers in PerioC individuals.

## Materials and methods

After approval by the Research Ethics Committee (number: 671855823.0.0000.5418), primary human gingival fibroblasts (HGFs) from Health and Grade C Periodontitis-affected subjects were selected from the PerioCells Biobank/UNICAMP. The cell populations used for the experiments were obtained in a previous study from the same research group, and the inclusion/exclusion criteria had already been described [32]. The bacterial stimulus used was a protein extract derived from *Aggregatibacter actinomycetemcomitans* (*Aa*PE). The null hypothesis of the study is that HGFs from patients presenting Grade C periodontitis and periodontally healthy individuals presents similar gene expression of some inflammasome markers, even after bacterial stimulation. So, to test this hypothesis, the following groups: Healthy Group (*n* = 5): HGFs from periodontally healthy (PH) individuals; and PerioC Group (*n* = 5): HGFs from Grade C periodontitis.

### Cell culture and bacterial challenge

To obtain the primary culture of HGFs, the protocol described by Silvério et al. 2010 was adopted [33]. Briefly, the gingival tissues were digested with 3 mg/ml of collagenase type 1 and 4 mg/ml of dispase (Gibco BRL, Life Technologies), and the cells were filtered through a 100 μm Cell Strainer. The suspension was centrifuged, and the cell pellet resuspended in standard culture medium, composed of DMEM, 10% FBS, 100 µg/mL Streptomycin, and 100 U/ml Penicillin (Gibco BRL, Life Technologies), seeded in 100 × 20 mm cell culture plates and incubated at 37 °C in an atmosphere saturated with 5% CO_2_ and 98% humidity. Cells between the third and fifth passages were used for all experiments.

JP2 strains of *Aa*, ATCC 29522, provided by FIOCRUZ/Manguinhos, were used. They were reactivated at 37 °C in an atmosphere saturated with 5% CO_2_ on agar plates containing Brain and Heart Infusion medium with Hemin and Vitamin K1 (Gibco BRL, Life Technologies). After 24 h of bacterial growth, the total protein was extracted as proposed by Albiero et al. 2017 [34]. Briefly, after the centrifugation of isolated colonies of *Aa* in 0.9% NaCl, the sediment was supplemented with 700 µL of ultrapure water and ≈ 0.16 g of 0.1 mm diameter zirconia beads and agitated in a Mini-BeadBeater (BioSpec Products) device and then centrifuged to obtain a beads-free supernatant, stored at −80 °C. The Bradford method (Bradford kit, Bio-Rad) was used to determine the total *Aa* protein extract concentration (*Aa*PE).

Cell viability, time course, and dose-response evaluation to *Aa*PE stimulation was previously tested in a fibroblast population of a PerioC subject, determining a concentration of 5 µg/mL, and 1.5 h of incubation [32]. Therefore, HGFs from 5 PH and 5 PerioC individuals were seeded in 100 × 20 mm cell culture plates, at a concentration of 90 × 10^4^ cells per plate and grown in standard medium for 24 h in a 37 °C and 5% CO_2_ environment. Then, the cells were challenged with *Aa*PE at 5 µg/mL, except on control plates, for 1.5 h. After this period, total RNA and DNA were collected, extracted, and treated as reported above.

Accordingly, the incubation period 1.5 h was selected based on previous optimization experiments demonstrating significant early transcriptional responses without compromising cell viability. This time enabled the investigation of immediate gene regulatory events associated with bacterial stimulation.

### RNA collection and gene expression

Total cell RNA was collected using TRIzol^®^ Reagent (Invitrogen™, ThermoFisher Scientific), extracted according to the manufacturers’ instructions and treated with deoxyribonuclease (DNase; DNA-free™, Ambion Inc.). The quality and concentration of RNA were measured using spectrophotometer (Nanodrop 2000, ThermoFisher Scientific), and a 10µL aliquot of the sample was used for the synthesis of complementary single-stranded DNA (cDNA; Roche Diagnostic Co.) in a final volume of 20µL. The quantitative polymerase chain reaction (qPCR) was performed using cDNA at a concentration of 25 ng/ml, the kit LightCycler 480 SYBR Green I Master (Roche Diagnostic Co.), with a pair of predefined human primers:

***18S*** **(housekeeping gene)**: 3′-CGGACAGGATTGACAGATTGATAGC-5′ (F); 5′-TGCCAGAGTCTCGTTCGTTATCG-3′ (R), ***TLR2***: 5’-CGTTCTCTCAGGTGACTGTC-3’ (F); 3’-CCTTTGGATCCTGCTTGC-5’ (R), ***TLR4***: 5’-CTCTCCTGCGTGAGACCAG-3’ (F); 3’- CCATGCATTGATAAGTAATATTAGGA-5’ (R), ***NLRP3***: 3’-GATCTTCGCTGCGATCAACA-5’ (F); 5’-GGGATTCGAAACACGTGCATTA-3’ (R), ***CASP1***: 5’-TGCCCACAGACATTCATACAGTTTC-3’ (F); 3’- GCCTGTTCCTGTGATGTGGAG-5’ (R), ***AIM2***: 5’-ATCTCCTGCTTGCCTTCTTGG-3’ (F); 3’-AAGTCTCTCCTCATGTTAAGCCTG-5’ (R), ***IL-10***: 5’-GCAGGGATGGAAGAAGAGAG-3’ (F); 3’-AGAGGGGACAGAAAGAGCAG-5’ (R), ***IL-1β***: 5’-CCATGTCCACCCAAGTCTCT-3’ (F); 3’-TGCTGGGCGGTAAAATTTCC-5’ (R), ***TNF-α***: 5’-TCCACCACCATATACTCCTCAC-3’ (F); 3’-CCTCCCAGATAGATGGGCTCA-5’ (R).

Samples were analyzed in triplicate, using water as a negative control. For relative gene expression, a determination was carried out using the cycle threshold (Ct) method and normalized to the 18S gene and calculated the expression of the target gene using the ΔCt formula.

### DNA collection and epigenetic analysis

Methylated/Hydroxymethylated DNA pattern of *IL-1β* and *NLRP3* genes was determined as previously described [35]. Briefly, to analyze DNA methylation and hydroxymethylation levels, genomic DNA was treated with T4-β-glucosyltransferase (T4-BGT) to add a glucose moiety to hydroxymethylated cytosines (5hmC), generating glucosylated 5hmC. The treated DNA was then divided into three groups: (1) digested with MspI, which is blocked by ghmC but not by methylated cytosines (mC) or 5hmC; (2) digested with HpaII, which is blocked by mC, 5hmC, and ghmC; and (3) undigested control samples. Digestions were conducted at 37 °C for 1 hour, with HpaII samples subjected to enzyme inactivation at 65 °C for 10 minutes. Following digestion, 40 ng of DNA from each group was amplified in triplicate by qPCR using primers targeting CCGG- containing regions of interest. The relative levels of methylation and hydroxymethylation were calculated using the cycle threshold (Ct) values from undigested controls as a reference. This method allowed the determination of the percentage of methylated alleles (HpaII-MspI/Control) and hydroxymethylated alleles (MspI/Control). The primers used for this analysis were: ***IL1β***: 5’-CGATGCACCTGTACGATCAC-3’(F); 3’-CATGGAGAATTAGCAAGCTGCC-5’ (R), and ***NLRP3***: 5’-GAGTTGCTCTTGTTGCCCAG-3’(F); 3’ CCTGAGGTCGGGAGTTTGAG-5’ (R).

## Statistical analysis and graphical presentation

Demographical and clinical data were compared between groups by Chi-square and Student’s t test. Gene expression and methylated pattern were compared by Mann-Whitney and Wilcoxon tests since the Shapiro-Wilk test showed no normal data distribution. A significance level of 5% was considered for all analyses.

## Results

**Fig. 1:**
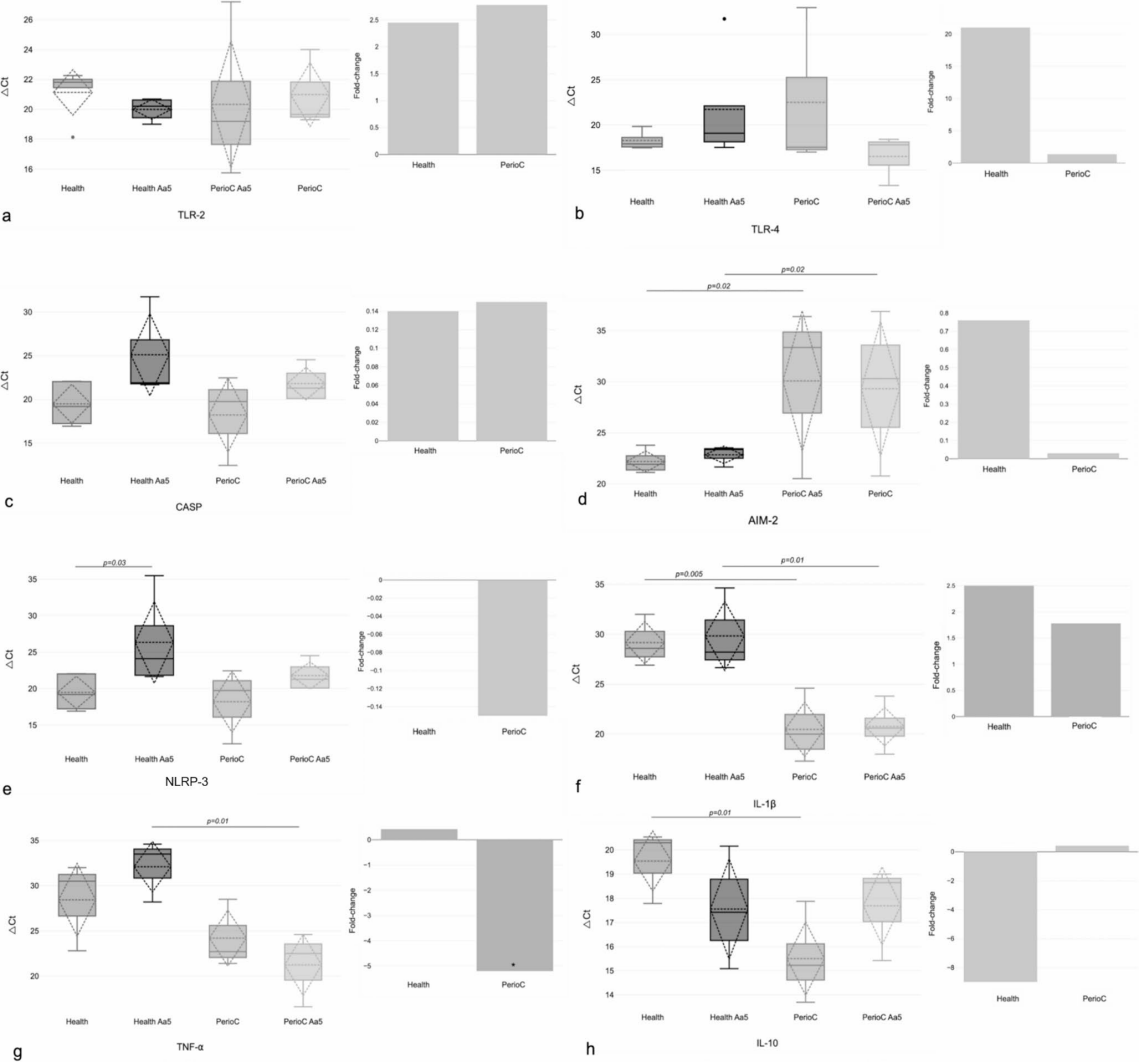
Relative mRNA levels of inflammasome-related markers. Figure 1**a**, **b**: Toll-like receptors 2 (*TLR2*) and 4 (*TLR4*); Fig. 1**c**-**e**: Caspase-1 (*CASP1*), Absent in Melanoma-2 (*AIM-2*), NOD-, LRR- and pyrin domain-containing protein 3 (*NLRP3*); Fig. 1**f**-**h**: and Interleukin-1β (*IL-1*β), *IL-10* and Tumor Necrosis Factor-*α* (*TNF-α*) in Periodontally healthy (Health) and Grade C Periodontitis (PerioC) individuals before and after 5 µg/mL of total protein Aa extract (*Aa*5). *Indicate a statistical difference between groups in fold-change analysis (Mann-Whitney test, p < 0.05)

Demographic and clinical information regarding cell donors is shown in Table 1. In Fig. 1, the relative mRNA levels of some inflammasome-related markers showed dissimilar fibroblast gene expression in PerioC compared to Healthy group. Before Aa stimulation, *TLR2* and *TLR4* (Fig. 1a, b) did not present differences between groups, as well as *CASP1*, *NLRP3* and *TNF-α* (Fig. 1c, e, g; *p* > 0.05). *AIM2* expression was higher in the PerioC group (Fig. 1d), than observed in *IL-1β* (Fig. 1f) and *IL-10* (Fig. 1h). The cell stimulation with total protein extract of *Aa* JP2 induced an increase in *NLRP3* expression in Healthy group, and not in PerioC. After *Aa*PE incubation, the *AIM2* levels were still higher in PerioC, while the levels of *NLRP3*, *IL-1β* and *TNF-α* were lower than Healthy group (*p* < 0.05).

**Table 1:** Clinical and demographical data of the study participants

	Health (*n* = 5)	PerioC (*n* = 5)
Age (years, average)	29.2	31.4
Sex (female/male)	4/1	4/1
Ethnicity (caucasian/african)	5/0	3/2
Plaque Index (%)	4.67	21.0
Bleeding on Probing (%)	3.88	37.0
Probing Pocket Depth (PPD) (%)	1.67	3.35
PPD ≥ 5 mm (%)	0	21.3

**Fig. 2:**
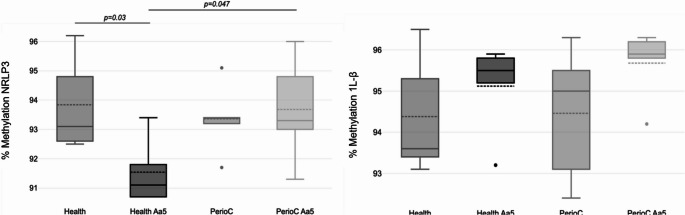
Methylated (%) DNA levels on *NLRP3* and *IL-1*β in Periodontally healthy (Health) and grade C periodontitis (PerioC) individuals before and after 5 µg/mL of total protein extract (*Aa*5)

*Aa* stimulation induced a reduction in the percentage of methylated NLRP3 DNA levels in Health but not in PerioC group (*p* > 0.05), resulting in a statistically significant difference between groups after stimulation (Fig. 2). Even though a trend in increasing the methylation percentage occurred in both groups in *IL-1*β gene, no significant difference was seen (*p* > 0.05).

## Discussion

Many studies report that the progression of periodontal disease is primarily related to bacterial infections and is associated with a complex interaction between pathogenic microorganisms and the host response [36, 37]. In this context, this interaction occurs through TLR2 and TLR4 membrane recognition receptors (PAMPs), which play a crucial role in innate immunity [10, 17, 19]. TLRs are essential for recognizing pathogens and activating the inflammatory pathway in periodontal disease by producing pro-inflammatory cytokines [24, 43, 44]. This process is crucial for defense against infection but can also lead to chronic inflammation and tissue destruction [17, 24, 31, 45]. Among the various pathways activated after antigen recognition, the inflammasome cascade is a major player in the periodontitis process.

Our results suggested an aberrant transcriptional response of inflammasome-related genes to the experimental conditions in PerioC HGFs. It could be observed a dysregulated inflammatory gene expression profile in PerioC fibroblasts, characterized by reduced expression of important inflammatory mediators such as IL-1β and TNF-α before and after bacterial stimulation. At the transcriptional level, this profile is more consistent with a hyporesponsive than a hyperresponsive phenotype, potentially reflecting impaired regulation of inflammatory pathways in these cells and these alterations may be associated with differences in local immune regulation and could potentially influence host responses observed in Grade C periodontitis [16, 21]. Inflammasome is a crucial component of the innate immune response. In this context, the reduced expression of IL-1β and TNF-α after *Aa* stimulation indicates a reduced transcriptional response to the bacterial challenge. TNF-α is a multifunctional cytokine that promotes inflammation and activates other immune pathways [46]. The marked increase in IL-1β in the healthy group suggests that an inflammatory response could be essential in the defense process. Importantly, IL-1β is the final product of the NLRP3 and AIM2 cascades, reflecting the transcriptional regulation of inflammasome-associated genes. However, one of the most intriguing findings is the lack of response in both groups for caspase-1, which is crucial for the maturation of pro-inflammatory cytokines such as IL-1β [20, 27, 38, 39].

Despite stimulation with *Aa*PE, there were no statistically significant differences in the expression of the TLR2 and TLR4 genes between HGFs from healthy patients and those with PerioC. These findings suggest that the inflammatory response mediated by these receptors may not be significantly altered by the PerioC disease or the exposure to *Aa*PE under experimental conditions. Thus, other mechanisms may be involved in regulating the inflammatory response in periodontal disease. In addition to membrane receptors TLR2 and TLR4, the NLRP3 inflammasome, a multiprotein complex, emerges as a primary regulator of macrophage-induced inflammation and strengthens the association between periodontitis and bacterial activators [11, 12, 16, 40, 41].

Recent studies show that the NLRP3 inflammasome constitutes a domain with nucleotide-binding properties from the leucine-rich repeat (NLR) family and the pyrin and HIN (PYHIN) domain families to form protein complexes [42]. The crucial function of the NLRP3 inflammasome is to activate caspase-1, which in turn leads to the maturation of IL-1β and induces pyroptosis [28, 29]. The importance of this key mediator has been widely studied in the immunity of human diseases, but its regulatory mechanism remains unclear in the literature [39]. According to our findings, there is an increase in the expression of NLRP3 in healthy HGFs exposed to *Aa*PE (5 µg/ml), suggesting upregulation of inflammasome-associated gene expression. This significant increase aligns with studies associating periodontal pathogens, such as *Aa*, with inflammasome activation, and the initiation of the inflammatory response [8, 24].

Meanwhile, HGFs from PerioC subjects did not show a change in NLRP3 gene expression after stimulation. Previous studies have shown that NLRP3 can contribute to persistent inflammation and tissue destruction in diseases such as periodontitis [24]. Under normal conditions, without inflammatory stimuli or pathogenic factors, NLRP3 expression remains at basal levels [43]. NLRP3 performs a surveillance function and is activated in situations of danger, such as in response to products derived from pathogens or signals of cellular damage [30, 44, 45]. Therefore, the increase in NLRP3 levels in healthy HGFs can be a positive sign for controlling and preventing the destruction of connective and bone tissue, confirming that the activation of inflammatory pathways such as NLRP3 is directly linked to the presence of pathogens [24, 43].

The increase in *NLRP3* expression in the PerioC group is supported by studies emphasizing the importance of maintaining inflammatory activity in normal and healthy tissues [43, 46]. This prevents the deregulation of this gene, which can lead to structural damage, including periodontitis. Another important signaling pathway of the NLRP3 inflammasome complex is the production of the caspase-1 gene, which plays a key role in tissue inflammation. Caspase-1 is responsible for cleaving cellular proteins, resulting in biochemical and morphological changes that lead to cell death. In this context, apoptosis is necessary for tissue homeostasis and repair [26, 27, 47].

Although the NLRP3 inflammasome and caspase-1 have interconnected functions in the inflammatory response, NLRP3 activation results in the liberation of caspase-1, which in turn cleaves pro-inflammatory cytokines such as IL-1β and IL-18 [20]. In some situations, this may occur alongside the initiation of apoptosis. In our results, the expression of the *CASP1* gene was similar in both groups, indicating that in environments of chronic inflammation, a complex modulation between apoptosis and inflammation occurs, serving as an important target for interventions.

It is important to emphasize that inflammasome activation is primarily regulated through post-translational mechanisms, including complex assembly and caspase-1 activation. Therefore, the mRNA expression data presented here should be interpreted as indicators of transcriptional regulation rather than direct evidence of inflammasome activation. Future studies incorporating protein quantification, caspase-1 activity assay, ASC (Apoptosis-associated Speck-like protein containing a CARD) speck formation, and IL-1β secretion analyses are necessary to confirm the functional relevance of the observed transcriptional changes.

A limitation of this study is the relatively small sample size. However, the use of primary human gingival fibroblasts obtained from well-characterized Grade C periodontitis patients inherently limits sample availability and is consistent with previous mechanistic studies in periodontal research. Although significant differences were identified, these findings should be considered exploratory and require confirmation in larger independent cohorts and complementary functional studies.

## Conclusion

Primary fibroblasts from patients with Grade C periodontitis demonstrated distinct inflammasome-related and epigenetic profiles compared with control cells after challenge with *A. actinomycetemcomitans* protein extract. Alterations in NLRP3 methylation and expression were associated with differences in the expression of inflammatory mediators, suggesting a possible link between epigenetic regulation and host immune responses in Grade C periodontitis. Further studies are needed to clarify the mechanisms underlying these associations.

## Data Availability

No datasets were generated or analysed during the current study.
